# HIGA: A Running History Information Guided Genetic Algorithm for Protein–Ligand Docking

**DOI:** 10.3390/molecules22122233

**Published:** 2017-12-15

**Authors:** Boxin Guan, Changsheng Zhang, Yuhai Zhao

**Affiliations:** Key Laboratory of Medical Image Computing of Northeastern University, Ministry of Education, and School of Computer Science and Engineering, Northeastern University, Shenyang 110819, China; 13940589067@sina.cn (B.G.); zhangchangsheng@ise.neu.edu.cn (C.Z.)

**Keywords:** running history information, drug design, genetic algorithm, protein-ligand docking

## Abstract

Protein-ligand docking is an essential part of computer-aided drug design, and it identifies the binding patterns of proteins and ligands by computer simulation. Though Lamarckian genetic algorithm (LGA) has demonstrated excellent performance in terms of protein-ligand docking problems, it can not memorize the history information that it has accessed, rendering it effort-consuming to discover some promising solutions. This article illustrates a novel optimization algorithm (HIGA), which is based on LGA for solving the protein-ligand docking problems with an aim to overcome the drawback mentioned above. A running history information guided model, which includes CE crossover, ED mutation, and BSP tree, is applied in the method. The novel algorithm is more efficient to find the lowest energy of protein-ligand docking. We evaluate the performance of HIGA in comparison with GA, LGA, EDGA, CEPGA, SODOCK, and ABC, the results of which indicate that HIGA outperforms other search algorithms.

## 1. Introduction

Drug molecular design, as a new drug research method and means, has achieved a lot of theoretical and practical research findings [1,2,3,4]. Protein-ligand docking is a typical method for structure-based drug discovery and design, the aim of which is to find the best ligand conformation of a ligand against a protein receptor target with the lowest energy [5,6,7,8,9]. The progress of X-ray diffraction technology of biological macromolecules provides us with more important structures of proteins and ligands. These structures can be used as targets for bioactive substances to control diseases in animals and plants, and they allow for people to understand the biological mechanisms of active substances simply [10,11,12]. The rapid development of computer technology has promoted the further development of molecular drug design effectively and significantly reduced the cost of drug research. For the docking process, the ligands are placed at the active site of the receptors, then the position and orientation of the ligands are adjusted by some binding and complementary principles, and then the optimal binding modes are obtained finally.

A search algorithm and a scoring function are the basic tools of a docking method for solving the two goals above. The scoring function is used to evaluate the binding conformation of ligands and receptors that were predicted by computer simulation [13,14,15,16,17]. In the docking process, it is necessary to obtain the binding affinity accurately as the basis for optimization. The scoring function not only provides a fast method for evaluating the binding affinity, but it also assists a docking in efficiently exploring the binding space of a ligand [18,19]. The score function can be directly used as the fitness function of optimization algorithms.

The search algorithm aims to identify the optimal binding mode between ligands and receptors, including the location of small ligands relative to proteins and conformational changes in molecules [20,21]. The best result of searching is the docked conformation with the lowest energy. The space search in molecular docking is the NP-hard problem, so it is impossible to traverse all of the search space. Heuristic algorithms have a lot of successful applications for protein-ligand docking problems [22,23,24]. For example, simulated annealing (SA) [25], genetic algorithm (GA) [26,27,28], Lamarckian genetic algorithm (LGA) [29], SODOCK [30,31,32], and artificial bee colony (ABC) [33]. However, the existing algorithms do not make a reasonable use of the history information, which results in the insufficient quality of the solutions that they obtain. Therefore, an efficient optimization algorithm that can find lower docking energy and RMSD is desirable.

The LGA is proven to be efficient, but it does not take advantage of the history information that is accumulated during the optimization procedure, resulting in it being hard to discover some promising solutions. As a result, we report an LGA-based novel genetic algorithm that enhances the performance of protein-ligand docking by utilizing the running history information in this article. The proposed algorithm can be abbreviated as HIGA, which stands for running history information guided genetic algorithm. Running history information refers to the information that retained during the running process of the algorithm, has a guiding role for the subsequent iterations. Running history information includes elite individuals, individuals with the historical optimal solution and suboptimal solution, and the location of individuals.

The three critical strengths of HIGA are as follows. (1) CE crossover is proposed to optimize crossover operation of the algorithm [34]. CE crossover uses history information to retain individuals with good genes, and these individuals make the subsequent population better; (2) ED muation is proposed to guide the evolutionary direction according to running historical information [35]; (3) Binary space partitioning (BSP) tree is employed to maintain the diversity of the individuals in a population [36]. The BSP tree can memorize all of the evaluated solutions so as to avoid solution re-evaluation.

The environment and the scoring function of AutoDock 4.2.6 are adopted as the experimental platform in the article [37,38,39]. AutoDock, as an open source academic software, which can embed the improved algorithm conveniently. AutoDock first uses the amino acid residues around the active site of the receptor to form a box. Then, it uses different types of atoms as the probe to scan, calculate the grid energy, and to search for the ligand in the range of the box. At last, it scores according to the different conformation, orientation, and position of the ligand. In order to demonstrate the power HIGA, we perform the experiement on a set of protein-ligand structures from PDBbind 2016 [40]. The performance of GA, LGA, EDGA, CEPGA, SODOCK, ABC, and HIGA is compared on these datasets. The experimental results show that our method has improved power in the aspects obtained energy and RMSD, convergence performance, data distribution, and hypothesis test.

## 2. Results and Discussion

### 2.1. Data Preparation and Parameter Setting

**Figure 1:**
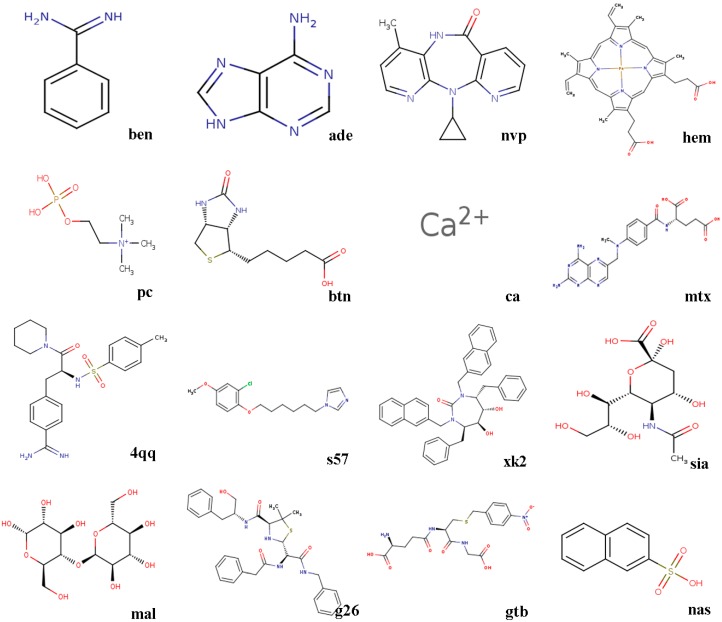
The molecular structures of ligands.

The docking power of seven algorithms is compared, and they are GA, LGA, EDGA, CEPGA, SODOCK, ABC, and HIGA. For the compared algorithms, the number of iterations is 27,000, the number of energy evaluations is 1.5 × 10^6^, the number of individuals is 50, and the other parameters are the default. The algorithms are terminated by reaching the number of iterations or the number of energy evaluations. We randomly choose a hundred X-ray crystallographic complexes (PDB) from PDBbind 2016 to make up Dataset 1, and then we choose sixteen complexes that have a different number of rotatable bonds in ligands from the hundred complexes to constitute Dataset 2. Before docking, we preprocess the downloaded proteins and ligands. The steps of protein processing are removing water molecules, adding charges, assigning hydrogens, and solvation. The procedures for treating ligands are adding charges, assigning hydrogens, detecting root, and choosing torsions. The molecular structures of the sixteen ligands in Dataset 2 are showed in Figure 1, and the sixteen complexes are briefly described below.

(1) 3ptb β-trypsin/ben (benzamidine)
  β-Trypsin, isolated from the pancreas of pigs, sheep, as well as cattle, is used as a protease. Benzamidine, an inhibitor, is generally utilized in suppressing proteolysis of proteins.
(2) 1aha α-momorcharin/ade (adenine)
  α-Momorcharin originates from seeds of Momordica charantia, while adenine is a biological component of organism.
(3) 3hvt HIV-1 reverse transcriptase/nvp
  HIV-1 reverse transcriptase (RT), a phosphate enzyme, is involved in synthesis of cDNA. Nvp is a strong, non-nucleoside RT suppressor.
(4) 1phg cytochrome P450-cam/hem (protoporphyrin IX)
  Cytochrome P450-cam, participating in metabolism of exogenous, as well as endogenous substances, is a superfamily of heme-thiolate proteins. Protoporphyrin IX, a purple brown crystalline powder, can dissolve in methanol, while is not soluble in ether, chloroform, acetone, or water.
(5) 2mcp McPC-603/pc (phosphocholine)
  McPC-603, a myeloma protein from mouse that binds to phosphocholine, interacts with phosphatidylcholine synthesis in tissues.
(6) 1stp streptavidin/btn (biotin)
  Streptavidin, a protein obtained from streptomyces, harbors a similar biological features with affinity. Biotin, a member of B vitamins, plays a critical role in normal metabolism of proteins as well as fats.
(7) 6rnt ribonuclease T1/ca (calcium ion)
  Ribonuclease T1, an endonuclease, is able to discard the non-hybridized RNA area in DNA-RNA hybrid. Calcium ion plays a vital role in human physiological functions.
(8) 4dfr dihydrofolate reductase/mtx (methotrexate)
  Dihydrofolate reductase, has universally been utilized as a therapeutic target in anti-tumor therapy, as well as other aspects. Methotrexate, a drug with potent immunosuppressive effect, is capable of inhibiting proliferation as well as division of immune cells.
(9) 1ett thrombin/4qq
  Thrombin is a formless, white to gray, freeze-dried powder, and 4qq is regarded as a non-polymer suppressor.
(10) 1hri human rhinovirus/s57
  Human rhinovirus causes the majority of human common cold. s57 is a member of imidazole.
(11) 1hvr protease/xk2
  Protease, an enzyme widely found in animals as well as plants, is capable of catalyzing protein catabolism. xk2, a small molecule inhibitor, is able to decrease or even prohibit chemical reaction rate.
(12) 4hmg hemagglutinin/sia (sialic acid)
  Hemagglutinin is the cause of coagulation of erythrocytes. Sialic acids, generated at terminal sugars, are members of acidic monosaccharides.
(13) 1cdg cyclodextrin glycosyl transferase/mal (maltose)
  Cyclodextrin glycosyltransferase, a bacterial enzyme, is able to produce cyclodextrins. Maltose, made of starch, as well as malt, is widely utilized as nutrient as well as culture medium.
(14) 1htf HIV-1 protease/g26
  HIV-1 protease is capable of separating newly-generated polyproteins into individual peptides. g26, a non-polymer suppressor, is an amide with easily oxidizable and highly reactive perssad.
(15) 1glq glutathione S-transferase/gtb (S-(P-nitrobenzyl)Glutathione)
  Glutathione S-transferase, a series of enzymes, is associated with hepatic detoxification process. S-(P-nitrobenzyl) Glutathione is a critical synthesis of glutathione precursor.
(16) 1tmn thermolysin/nas (2-naphthalenesulfonic acid)
  Thermolysin, a biological component, is featured by a more rapid hydrolysis of hydrophobic amino acids. 2-naphthalenesulfonic acid, white powder or crystal, can dissolve in water but not in alcohol, which is widely adopted in organic synthesis.

### 2.2. Comparison of Energy and RMSD

The primary objective of our experiment is to find the lowest energy. The values of the lowest energy, calculated by the semi-empirical free energy force field [15], is the most important criterion to evaluate the performance of the compared algorithms. Root-mean-square positional deviation (RMSD) is also the commonly used standard to evaluate the molecular docking results. RMSD compares the optimal docking structure with the experimentally measured actual structure. If the RMSD is smaller than a given threshold 2.0 Å after docking, then the docking can be considered successful. Each algorithm runs one time in Dataset 1. The success of the docking is recorded, Average RMSD (all cases) and Average RMSD (RMSD < 2 Å) are calculated (Table 1). For the number of success cases and the Average RMSD, HIGA is obviously superior to other algorithms. Each algorithm runs twenty times in Dataset 2, the lowest energy and RMSD are recorded, and the results are showed in Table 2. For the lowest energy of the sixteen complexes, HIGA finds the twelve lowest values, EDGA finds the two lowest values, CEPGA and SODOCK find the one lowest value respectively, and GA, LGA, ABC do not find any of the lowest values. Although HIGA does not find the lowest energy in 1aha, 1ett, 4hmg, and 1htf, the energy values found are still promising. For example, the energy −16.15 kcal mol^−1^ found by HIGA is close the lowest energy −16.23 kcal mol^−1^ found by EDGA in 1aha; the energy −21.17 kcal mol^−1^ found by HIGA is close the lowest energy −21.79 kcal mol^−1^ found by SODOCK in 1htf. The number of the lowest RMSD that is found by HIGA, GA, LGA, EDGA, CEPGA, ABC SODOCK is 7, 2, 1, 2, 1, 1 and 2, respectively. HIGA has no absolute advantage in finding the lowest RMSD, but it is better than the other algorithms. In conclusion, the best search method is HIGA with regard to its average performance.

### 2.3. Cluster Analysis of Docked Conformations

After twenty times docking of each complex in Dataset 2, twenty docked conformations are obtained. These conformations exhaustively compared to one another to determine similarities, and they are clustered if they are similar enough. The range of the formed clusters is 0 to 20. The clusters are ranked in order of increasing energy, by the lowest energy in each cluster. Rank 1 is the lowest energy cluster, it refers to how often the structure with the lowest energy is found. The concentration of the clusters and the docked structures in rank 1 can reflect the stability of the algorithm. The cluster analysis is performed in Dataset 2, and the results are shown in Table 3. The mean of the number of clusters found is lowest for HIGA (3.72), followed by CEPGA (4.04), EDGA (4.24), LGA (4.58), SODOCK (10.30), ABC (6.52), and finally GA (13.04). The mean of the number of docked structures in rank 1 is 17.04 for HIGA, 16.20 for CEPGA, 15.92 for EDGA, 15.72 for LGA, 11.82 for SODOCK, 8.70 for ABC, and 8.00 for GA. Hence, the most reliable search method is HIGA.

### 2.4. Convergence Analysis

**Figure 2:**
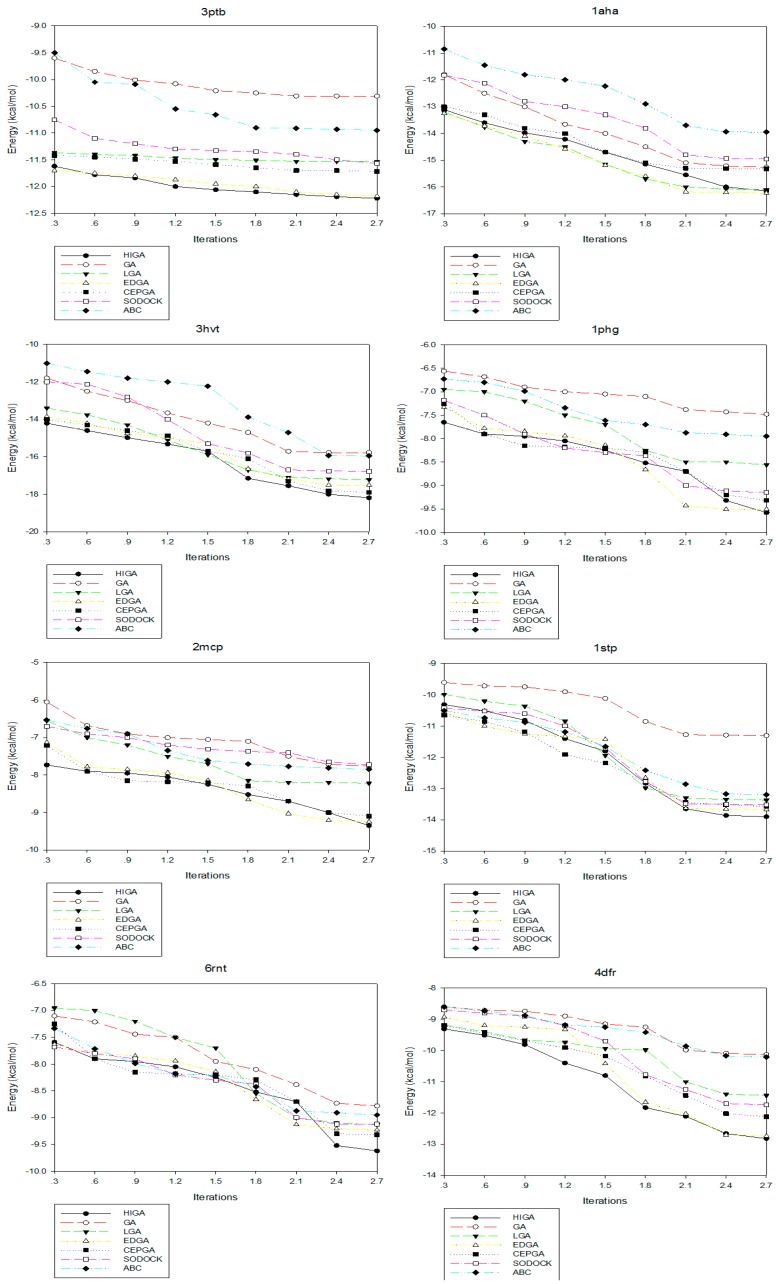

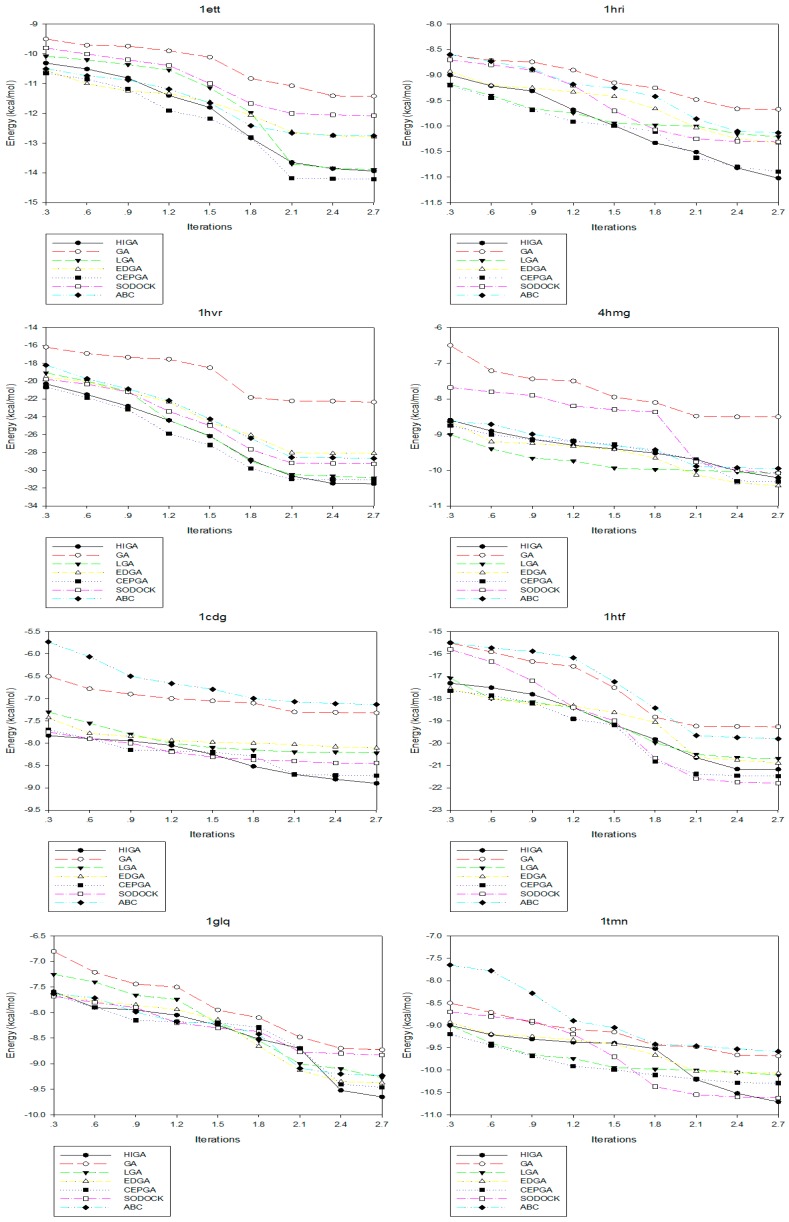
Convergence diagrams of seven algorithms in different X-ray crystallographic complexes (PDB).

Convergence means that the convergence curve of the objective solution tends to be stable after several iterations. The convergence diagrams of seven different algorithms for solving different test cases of Dataset 2 are shown in Figure 2. The number of iterations is 3000, 6000, 9000, 12,000, 15,000, 18,000, 21,000, 24,000 and 27,000, respectively, and these values are used as the horizontal axis of the convergence diagrams. The energy of each algorithm under different iteration times is calculated as the vertical axis. At the early stage of each algorithm, the energy value decreases as the number of iterations increases. But, in the later stage, the energy values of some algorithms tend to be fixed. This phenomenon, which is caused by the decrease of population diversity and the loss of evolutionary capacity, is called premature convergence. For example, LGA, EDGA, and SODOCK are prematurely convergent after iterating 15,000 times in 2mcp; GA and ABC are prematurely convergent after iterating 18,000 times in 2mcp; LGA, EDGA, SODOCK, and ABC are prematurely convergent after iterating 21,000 times in 6rnt. From these graphs, it can be concluded that HIGA is superior to other algorithms regarding preventing premature and solution quality.

### 2.5. Data Distribution Analysis

**Figure 3:**
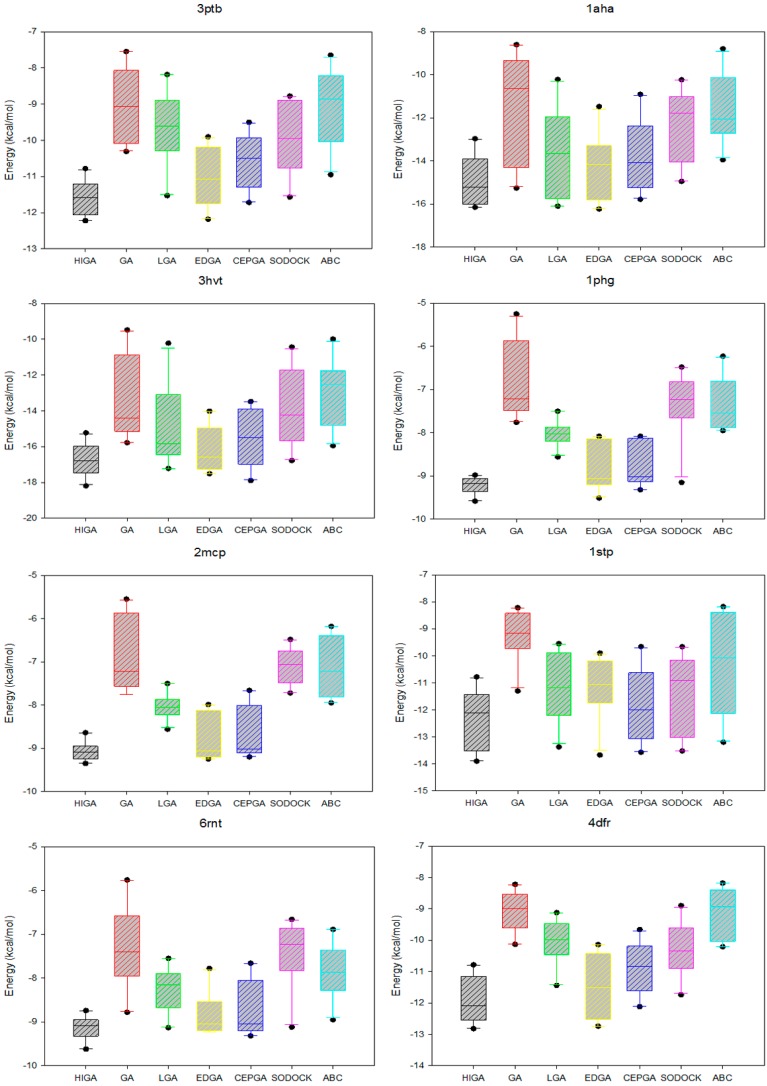

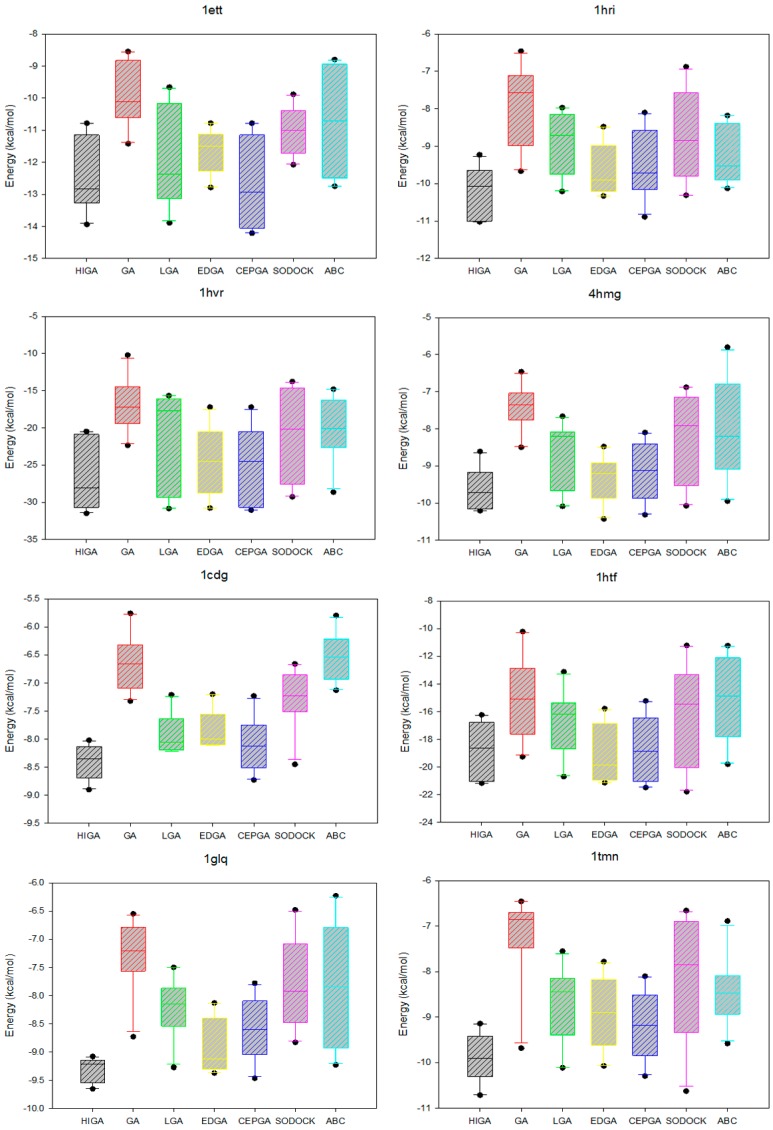
Box plots of seven algorithms in different PDB.

The data distribution can reflect the concentration of the data and the stability of the algorithm. We calculate the minimum, the first quartile, the median, the third quartile, and the maximum of the energy values of each PDB, and then we use the five statistical quantities to draw the box plots (Figure 3). The median, which is not affected by the extreme data, is suitable as a centralized trend value. It is evident that the median energy of HIGA is lower than that of the other algorithms. The first quartile is the upper boundary of the box, the third quartile is the lower boundary of the box, and the data distribution is concentrated or dispersed and can be determined by observing the box. It can be seen that the data distribution of HIGA is the most concentrated. The points outside the maximum and minimum are the outliers, and the outliers have an undesirable consequence of data distribution. For example, the outlier of GA in 1glq; the outlier of LGA in 3hvt; the outlier of EDGA in 1stp; the outlier of SODOCK in 1cdg; the outlier of ABC in 4hmg. HIGA and CEPGA have no outliers. It can be concluded that HIGA is a stable method for protein-ligand docking.

### 2.6. Execution Time Analysis

The execution time of the seven compared algorithms for solving different complexes of Dataset 2 are shown in Table 4. The time is recorded by how many seconds per run. There a direct relation between the execution time and the problem complexity. In addition to a few complexes, the execution time of the algorithms increases as the number of rotatable bonds increases. As seen from the table, the execution time of GA is the fastest, followed by LGA, EDGA, CEPGA, HIGA, ABC, and SODOCK. The best performance algorithm HIGA in previous experiments does not play an advantage in the time performance. However, the execution time of HIGA is not greatly increased when compared to the fastest algorithm GA. This can demonstrate that HIGA does not raise the performance at the cost of increasing the execution time.

### 2.7. Comparison Based on the Hypothesis Test

We use the hypothesis test to determine the difference between each algorithm in Table 5. Five of the best values obtained by each algorithm for every PDB are taken, and the critical value of hypothesis test is set to 0.05. When comparing algorithm A to algorithm B, we can conclude the algorithm A is significantly different from the algorithm B if the *p*-value is less than 0.05. The significant difference demonstrates that the algorithm A is superior to the algorithm B. As seen from the table, HIGA, EDGA, and CEPGA are better than GA, LGA, SODOCK, and ABC in most of the PDBs. Furthermore, HIGA is better than EDGA and CEPGA according to the *p*-value. We can make a conclusion from the statistical analysis that HIGA is significantly better than the other algorithms.

## 3. Materials and Methods

### 3.1. Running History Information Guided Genetic Algorithm

**Figure 4:**
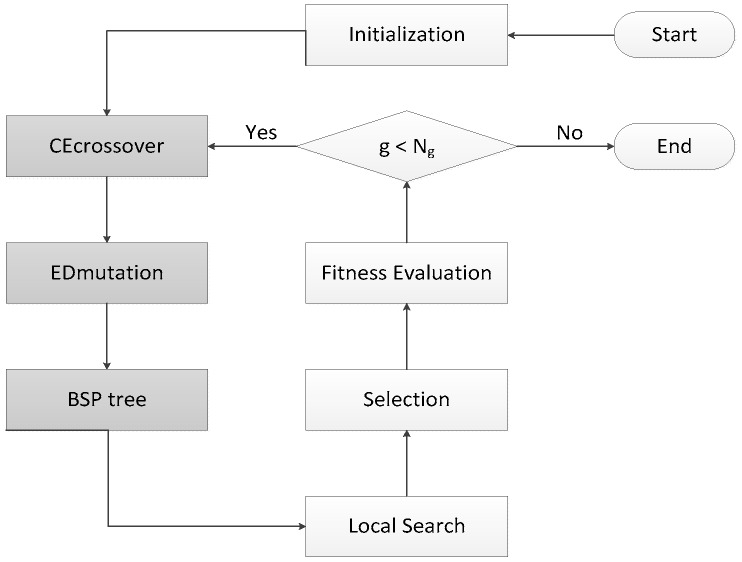
Block diagram of HIGA.

HIGA is an LGA-based hybrid search algorithm that is designed for protein-ligand docking. GA is an algorithm for simulating natural evolution process, and it generates solutions to optimise problems with the assistance of mutation, selection, and crossover. In each iteration of the genetic algorithm, the individual selection is made according to the fitness of the individual in the feasible region of the problem. Thus, a new and better approximate solution is generated. GA is simple, easy to realize, and has high robustness, so it has been successfully applied to solve the protein-ligand docking problem. LGA, which combines GA with local search method, searches the potential energy surface rapidly by GA and optimises the potential energy surface by local search method. LGA is proved to be an effective method for the protein-ligand docking, but the solutions that are obtained by LGA are unstable because of its randomness. Therefore, HIGA is proposed to overcome the drawback. Three core mechanisms are used to enhance the performance of HIGA for docking problems, and they are CE crossover, ED muation, and binary space partitioning (BSP) tree. CE crossover uses history information to improve the randomness of crossover operation. ED muation can guide the solutions to evolve in a better direction. BSP tree can memorize evaluated solutions that it has visited so that the diversity of solutions is maintained and the waste of computer resources is reduced. Taking the three techniques into consideration, the novel algorithm is guided to find some promising solutions by the running history information. Figure 4 is the block diagram of HIGA, and the grey parts are the three innovative mechanisms, which are newly added into LGA. HIGA first initializes the population randomly, and then obtains the next population by CE crossover, ED mutation, BSP tree, local search, selection and fitness evaluation. The process is iterated until a preset termination condition is reached.

### 3.2. CE Crossover

Since the parents of elite individuals are not preserved in the crossover process of LGA, the chances of getting better solutions are reduced by the subsequent crossover operation. CE crossover is proposed to solve the problem in this section. This new crossover can preserve the parents of the elite individuals with the best solution so that the good genes can be extended to improve the quality of the next-generation population. Table 6 shows the pseudocode of CE crossover. In the strategy, M_father_ and M_mother_ are introduced, in which M_father_ and M_mother_ represent the parents of the elite individual, e. The individual with current optimum fitness is selected as the elite individual at current iteration, and M_father_ and M_mother_ are saved. The preserved individuals and the individuals of next-generation form a new population. The genes of these preserved individuals are excellent, and the possibility that they continue to reproduce individuals with good genes is greater. For each iteration, the number of elite individuals is set up to a certain percentage of the number of individuals, and the default is ten percent.

### 3.3. ED Mutation

In LGA, some genes of the individuals are random changes by mutation operation, which results in the search direction of the algorithm is also random and aimless. At the early stage of this algorithm, the randomness plays a very positive guiding effect for global search. It is because the optimal solution in which the direction is unknown in the case of no search experience. With the continuous iteration of the algorithm, the group search experience is accumulating, the direction is gradually clear, and the search space starts to converge gradually. This means that the current best solution is inevitably abandoned, even the global best solution. Therefore, ED mutation is proposed to optimize the search direction. The pseudocode of ED mutation is shown in Table 7. Where m_i_ is the current solution; θ and δ are random numbers; β is a particular adjustable parameter, M_optimum_ is the historic optimal solution; and, M_sub_ represents is the historic suboptimal solution; M_max_ is the maximum solution; M_min_ is the minimum solution. In the formula, the historic optimal solution and the historic suboptimal solution are used to ensure the correctness of the optimal solution direction. This mechanism is similar to the addition of vectors, and the addition can ensure that the algorithm evolves towards a better direction.

### 3.4. BSP Tree

The BSP tree that has been applied in non-revisiting genetic algorithm (NrGA) is used to store the evaluated solutions, and it divides the search space in accordance with the cumulative distribution of the solutions being evaluated. The BSP tree has only one root node at the beginning, and this node represents the entire search space. Each node that is subsequently inserted into BSP tree represents a subspace. If a parent node has two child nodes (l and r), the subspaces that are represented by the two child nodes are disjoint, and the sum of the two spaces is the subspace corresponding to the parent node. BSP tree is different from with Octree employed in QuickVina-W [41]. BSP tree stores all of the solutions that the algorithm has searched before, while Octree stores high-quality history points which are the output of last iteration of local optimization from all of the searching threads during the runtime. Table 8 shows the pseudo code for the working principle of BSP tree. Where RF is the revisit flag; and, d is the distance between two nodes. The position of each previous individual generated by the algorithm is recorded in a node of the tree. When the new generated individual m visits the node l or r, their positions are checked. If m = l or m = r, m is revisited and RF is 1. If the solution is revisited, it mutates to a nearest unvisited neighbor subspace. BSP tree can guarantee the location of all solutions is different so that the diversity of the individuals is maintained and the sample space of GA is increased.

## 4. Conclusions

The article presents HIGA, which combines binary space partitioning (BSP), ED muation, and CE crossover to extend the power of the LGA-based algorithm. CE crossover amplifies the probability of repeated use of the individual with good genes, so that HIGA is more suitable to the changes of the environment evolution. ED mutation provides a guarantee for the evolutionary direction of HIGA, and its concept originates from the property of vector addition. By using the BSP tree, the search algorithm can not only remove revisits, but also guide to search for the next unvisited position. We have compared the performance of HIGA, GA, LGA, EDGA, CEPGA, SODOCK, and ABC, the results of which indicate that HIGA outperforms that the other algorithms, suggesting that HIGA can enhance the power of AutoDock to protein-ligand docking. 

## Figures and Tables

**Table 1:** Results of success case and average root-mean-square positional deviation (RMSD).

Algorithm	Success Case	Average RMSD (All Cases)	Average RMSD (RMSD < 2 Å)
HIGA	90	1.81	1.32
GA	58	3.12	1.80
LGA	68	2.55	1.69
EDGA	77	2.21	1.62
CEPGA	81	2.13	1.59
SODOCK	73	2.88	1.79
ABC	62	3.25	1.83

**Table 2:** The lowest energy and RMSD results.

			HIGA		GA		LGA		EDGA		CEPGA		SODOCK		ABC	
PDB	Ligand	Torsions	Energy	RMSD	Energy	RMSD	Energy	RMSD	Energy	RMSD	Energy	RMSD	Energy	RMSD	Energy	RMSD
3ptb	ben	0	−12.22	1.95	−10.31	1.66	−11.53	1.92	−12.18	1.95	−11.72	1.90	−11.57	2.00	−10.95	1.97
1aha	ade	1	−16.15	0.90	−15.26	1.20	−16.10	0.45	−16.23	0.38	−15.32	0.89	−14.95	1.44	−13.95	1.85
3hvt	nvp	2	−18.19	0.45	−15.78	0.40	−17.22	0.33	−17.52	0.53	−17.90	0.30	−16.78	0.58	−15.95	0.68
1phg	hem	3	−9.58	0.60	−7.48	1.25	−8.56	0.80	−9.51	0.70	−9.32	0.64	−9.15	1.34	−7.95	1.67
2mcp	pc	4	−9.35	1.15	−7.76	1.46	−8.22	1.33	−9.25	1.23	−9.10	1.20	−7.72	1.42	−7.85	1.64
1stp	btn	5	−13.90	0.85	−11.30	1.84	−13.37	1.65	−13.67	1.25	−13.57	0.90	−13.52	1.00	−13.20	1.58
6rnt	ca	6	−9.62	0.50	−8.78	0.65	−9.13	0.70	−9.23	0.85	−9.32	0.58	−9.12	1.95	−8.95	1.45
4dfr	mtx	7	−12.82	1.56	−10.13	0.90	−11.44	1.23	−12.74	1.59	−12.12	1.90	−11.74	1.67	−10.21	1.97
1ett	4qq	8	−13.94	1.40	−11.42	1.62	−13.89	1.38	−12.79	1.20	−14.21	1.29	−12.08	1.54	−12.75	1.65
1hri	s57	9	−11.02	1.18	−9.67	1.80	−10.21	1.87	−10.33	1.37	−10.89	1.38	−10.31	1.68	−10.13	1.67
1hvr	xk2	10	−31.50	0.80	−22.35	1.08	−30.85	0.62	−28.10	0.75	−31.06	0.64	−29.29	0.68	−28.65	0.85
4hmg	sia	11	−10.21	1.65	−8.50	1.68	−10.09	1.70	−10.43	1.58	−10.32	1.89	−10.08	1.36	−9.95	1.60
1cdg	mal	12	−8.90	1.65	−7.32	1.69	−8.22	1.94	−8.11	1.54	−8.73	1.48	−8.45	1.80	−7.13	1.12
1htf	g26	13	−21.17	1.20	−19.26	1.58	−20.69	1.33	−20.89	1.30	−21.48	1.27	−21.79	1.42	−19.80	1.80
1glq	gtb	14	−9.65	1.25	−8.73	1.27	−9.27	1.87	−9.37	1.60	−9.46	1.38	−8.83	1.90	−9.23	1.58
1tmn	nas	15	−10.71	0.95	−9.68	1.11	−10.11	1.20	−10.07	1.45	−10.29	0.85	−10.62	1.95	−9.58	0.65

**Table 3:** Results of the rate of clusters and the rate in rank 1.

Algorithm	HIGA	GA	LGA	EDGA	CEPGA	SODOCK	ABC
Number of clusters	3.72	14.00	4.58	4.24	4.04	10.30	13.04
Number in rank 1	17.04	8.00	15.72	15.92	16.20	11.82	8.70

**Table 4:** Results of execution time.

PDB	Torsions	HIGA	GA	LGA	EDGA	CEPGA	SODOCK	ABC
3ptb	0	1.79	1.56	1.74	1.77	1.75	1.89	1.90
1aha	1	1.91	1.62	1.80	1.83	1.90	1.95	2.08
3hvt	2	1.97	1.75	1.93	1.95	1.96	1.94	2.13
1phg	3	2.75	2.21	2.55	2.61	2.70	2.23	2.65
2mcp	4	2.72	2.35	2.62	2.70	2.68	2.51	2.69
1stp	5	3.79	3.01	3.53	3.77	3.59	3.84	3.81
6rnt	6	3.84	3.12	3.14	3.70	3.54	4.27	3.64
4dfr	7	5.47	4.98	5.18	5.33	5.45	4.99	6.06
1ett	8	8.44	7.83	8.17	8.28	8.37	8.29	7.97
1hri	9	10.49	10.15	10.37	10.39	10.41	12.34	11.06
1hvr	10	12.53	12.07	12.13	12.28	12.51	14.86	11.92
4hmg	11	13.90	13.21	13.84	13.95	13.89	16.09	13.60
1cdg	12	12.71	12.38	12.59	12.61	12.66	15.92	13.06
1htf	13	13.01	12.49	12.78	12.99	12.84	16.26	13.18
1glq	14	15.87	14.95	15.50	15.77	15.85	20.12	17.22
Average		7.41	6.91	7.19	7.33	7.34	8.5	7.53

**Table 5:** Results of hypothesis test.

PDB		HIGA	GA	LGA	EDGA	CEPGA	SODOCK	ABC
3ptb	HIGA	-	0.004	0.010	0.044	0.035	0.012	0.008
	GA	0.996	-	0.992	0.995	0.994	0.993	0.688
	LGA	0.990	0.008	-	0.986	0.982	0.563	0.425
	EDGA	0.956	0.005	0.014	-	0.340	0.017	0.010
	CEPGA	0.965	0.006	0.018	0.660	-	0.028	0.011
	SODOCK	0.988	0.007	0.437	0.983	0.972	-	0.306
	ABC	0.992	0.312	0.575	0.990	0.989	0.694	-
1aha	HIGA	-	0.029	0.062	0.524	0.044	0.024	0.017
	GA	0.971	-	0.964	0.988	0.905	0.342	0.260
	LGA	0.938	0.036	-	0.981	0.460	0.030	0.024
	EDGA	0.476	0.012	0.019	-	0.017	0.010	0.005
	CEPGA	0.956	0.095	0.540	0.983	-	0.038	0.027
	SODOCK	0.976	0.658	0.970	0.990	0.962	-	0.470
	ABC	0.983	0.740	0.976	0.995	0.973	0.530	-
3hvt	HIGA	-	0.005	0.017	0.020	0.023	0.012	0.008
	GA	0.995	-	0.795	0.974	0.993	0.788	0.537
	LGA	0.983	0.205	-	0.878	0.965	0.324	0.208
	EDGA	0.980	0.026	0.122	-	0.515	0.103	0.033
	CEPGA	0.977	0.007	0.035	0.485	-	0.029	0.011
	SODOCK	0.988	0.212	0.676	0.897	0.971	-	0.215
	ABC	0.992	0.463	0.792	0.967	0.989	0.785	-
1phg	HIGA	-	0.006	0.015	0.242	0.046	0.023	0.010
	GA	0.994	-	0.682	0.991	0.983	0.968	0.545
	LGA	0.985	0.318	-	0.976	0.962	0.620	0.422
	EDGA	0.758	0.009	0.024	-	0.440	0.038	0.015
	CEPGA	0.954	0.017	0.038	0.560	-	0.045	0.028
	SODOCK	0.977	0.032	0.380	0.962	0.955	-	0.240
	ABC	0.990	0.455	0.578	0.985	0.972	0.760	-
2mcp	HIGA	-	0.002	0.008	0.036	0.015	0.001	0.004
	GA	0.998	-	0.792	0.994	0.992	0.492	0.610
	LGA	0.992	0.208	-	0.988	0.987	0.092	0.224
	EDGA	0.964	0.006	0.012	-	0.442	0.005	0.011
	CEPGA	0.985	0.008	0.013	0.558	-	0.007	0.012
	SODOCK	0.999	0.508	0.908	0.995	0.993	-	0.640
	ABC	0.996	0.390	0.776	0.989	0.988	0.360	-
1stp	HIGA	-	0.001	0.006	0.048	0.033	0.014	0.003
	GA	0.999	-	0.791	0.964	0.952	0.892	0.587
	LGA	0.994	0.209	-	0.942	0.887	0.624	0.450
	EDGA	0.952	0.036	0.058	-	0.414	0.082	0.043
	CEPGA	0.967	0.048	0.113	0.586	-	0.208	0.062
	SODOCK	0.986	0.108	0.376	0.918	0.792	-	0.215
	ABC	0.997	0.413	0.550	0.957	0.938	0.785	-
6rnt	HIGA	-	0.005	0.020	0.022	0.038	0.015	0.007
	GA	0.995	-	0.942	0.965	0.985	0.695	0.588
	LGA	0.980	0.058	-	0.792	0.888	0.368	0.127
	EDGA	0.978	0.035	0.208	-	0.504	0.059	0.040
	CEPGA	0.962	0.015	0.112	0.496	-	0.047	0.025
	SODOCK	0.985	0.305	0.632	0.941	0.953	-	0.404
	ABC	0.993	0.412	0.873	0.960	0.975	0.596	-
4dfr	HIGA	-	0.002	0.006	0.035	0.032	0.021	0.003
	GA	0.998	-	0.961	0.993	0.985	0.972	0.587
	LGA	0.994	0.039	-	0.966	0.950	0.624	0.450
	EDGA	0.965	0.007	0.034	-	0.490	0.042	0.013
	CEPGA	0.968	0.015	0.050	0.510	-	0.060	0.016
	SODOCK	0.979	0.028	0.376	0.958	0.940	-	0.215
	ABC	0.997	0.413	0.550	0.987	0.984	0.785	-
1ett	HIGA	-	0.008	0.082	0.060	0.515	0.044	0.057
	GA	0.992	-	0.986	0.925	0.998	0.562	0.637
	LGA	0.918	0.014	-	0.482	0.932	0.073	0.151
	EDGA	0.940	0.075	0.518	-	0.950	0.077	0.205
	CEPGA	0.485	0.002	0.068	0.050	-	0.040	0.045
	SODOCK	0.956	0.432	0.927	0.923	0.960	-	0.520
	ABC	0.943	0.363	0.849	0.795	0.955	0.480	-
1hri	HIGA	-	0.001	0.018	0.025	0.041	0.021	0.004
	GA	0.999	-	0.976	0.997	0.998	0.982	0.635
	LGA	0.982	0.024	-	0.862	0.890	0.723	0.117
	EDGA	0.975	0.003	0.138	-	0.504	0.140	0.015
	CEPGA	0.959	0.002	0.110	0.406	-	0.130	0.010
	SODOCK	0.979	0.018	0.277	0.860	0.870	-	0.020
	ABC	0.996	0.365	0.883	0.985	0.990	0.980	-
1hvr	HIGA	-	0.002	0.030	0.012	0.045	0.021	0.014
	GA	0.998	-	0.995	0.562	0.996	0.942	0.665
	LGA	0.970	0.005	-	0.026	0.957	0.177	0.044
	EDGA	0.988	0.438	0.974	-	0.982	0.711	0.609
	CEPGA	0.955	0.004	0.043	0.018	-	0.035	0.024
	SODOCK	0.979	0.058	0.823	0.289	0.965	-	0.368
	ABC	0.986	0.335	0.956	0.391	0.976	0.632	-
4hmg	HIGA	-	0.022	0.072	0.522	0.514	0.054	0.032
	GA	0.978	-	0.972	0.995	0.985	0.928	0.900
	LGA	0.928	0.028	-	0.980	0.945	0.417	0.214
	EDGA	0.478	0.005	0.020	-	0.487	0.017	0.010
	CEPGA	0.486	0.015	0.045	0.513	-	0.042	0.026
	SODOCK	0.946	0.072	0.583	0.983	0.958	-	0.240
	ABC	0.968	0.100	0.786	0.990	0.974	0.760	-
1cdg	HIGA	-	0.003	0.021	0.014	0.037	0.032	0.001
	GA	0.997	-	0.883	0.784	0.995	0.985	0.408
	LGA	0.979	0.117	-	0.483	0.952	0.763	0.105
	EDGA	0.986	0.216	0.517	-	0.983	0.844	0.125
	CEPGA	0.963	0.005	0.048	0.017	-	0.059	0.003
	SODOCK	0.968	0.015	0.237	0.156	0.941	-	0.012
	ABC	0.999	0.592	0.895	0.875	0.997	0.988	-
1htf	HIGA	-	0.015	0.243	0.480	0.544	0.624	0.030
	GA	0.985	-	0.973	0.974	0.985	0.987	0.637
	LGA	0.753	0.027	-	0.652	0.759	0.883	0.251
	EDGA	0.520	0.016	0.348	-	0.618	0.640	0.235
	CEPGA	0.456	0.015	0.241	0.382	-	0.538	0.028
	SODOCK	0.376	0.013	0.127	0.360	0.462	-	0.017
	ABC	0.970	0.363	0.749	0.765	0.972	0.983	-
1glq	HIGA	-	0.001	0.012	0.018	0.022	0.002	0.005
	GA	0.999	-	0.982	0.991	0.995	0.695	0.788
	LGA	0.988	0.018	-	0.955	0.965	0.163	0.227
	EDGA	0.982	0.009	0.045	-	0.510	0.039	0.042
	CEPGA	0.978	0.005	0.035	0.490	-	0.027	0.031
	SODOCK	0.998	0.305	0.837	0.961	0.973	-	0.704
	ABC	0.995	0.212	0.773	0.958	0.969	0.296	-
1tmn	HIGA	-	0.004	0.023	0.012	0.028	0.043	0.002
	GA	0.996	-	0.783	0.692	0.965	0.983	0.408
	LGA	0.977	0.217	-	0.283	0.716	0.746	0.105
	EDGA	0.988	0.308	0.717	-	0.810	0.975	0.125
	CEPGA	0.972	0.035	0.284	0.190	-	0.644	0.028
	SODOCK	0.953	0.017	0.256	0.025	0.356	-	0.012
	ABC	0.998	0.592	0.895	0.875	0.972	0.988	-

**Table 6:** Pseudocode of CE crossover.

Algorithm: CE Crossover
Input: (1) a population with *n* indiviudals, (2) elitists *e*.
Output: a population after CE crossover
01. **For** *i*: = *1* to *n* **do**
02. Find the historial optimal individual *m_0_*
03. **If** the fitness of current individual *m_i_* < *m_0_* **then**
04. *m_0_* = *m_i_*
05. e = *m_0_*
06. preserve *M_father_* and *M_mother_*
07. **End if**
08. *M_father_*, *M_mother_ and e* ⊂ next population
09. **End for**

**Table 7:** Pseudocode of ED mutation.

Algorithm: ED Mutation
Input: (1) a population with *n* indiviudals, (2) balance factor *β*.
Output: a population after ED mutation
01. **For** *i*: = *1* to *n* **do**
02. Find the optimal solution *M_optimum_* and the historic suboptimal solution *M_sub_*
03. **If** *θ* < *β* **then**
04. *m_i_* = *m_min_* + *θ* (*M_ma_*_x_ − *M_min_*)
05. **Else**
06. *m_i_* = *m_i_* + *θ* (*M_optimum_* − *M_sub_*) +δ(*M_optimum_* − *m_i_*)
07. **End if**
08. **End for**

**Table 8:** Pseudocode of BSP tree.

Algorithm: BSP Tree
Input: (1) an individual *m*, (2) BSP tree *T* (3) revisit flag *RF*
Output: an individual *m* that never revisits
01. *Curr_node*: = *root node* of *T*
02. *RF* = *0*
03. **If** (Curr_node has two child nodes: *l* and *r*) **then**
04. Compare *m* with child node *l* and *r*
05. **If** (*m* = *l*) or (*m* = *r*) **then**
06. *RF* = *1*
07. **End if**
08. **If** *d* (*l*, *m*) < *d* (*r*, *m*) **then**
09. *Curr_node*: = child node *l*
10. **Else**
11. *Curr_node*: = child node *r*
12. **End if**
13. **Repeat steps 03-12**
14. **Else**
15. **If** (*RF* = *0*) **then**
16. Insert a child node to *Curr_node* that records *m*
17. **Else**
18. Creat a new child node by mutating
19. **End if**
20. **End if**
